# Urinary Bladder Carcinosarcoma (Sarcomatoid Carcinoma) With Long Survival After Transurethral Resection: A Case Report

**DOI:** 10.7759/cureus.59992

**Published:** 2024-05-09

**Authors:** Ahmed M Badheeb, Omar Alkhanbashi, Shehab S Al Hammadi, Faisal Ahmed, Hasan Guzailan, Omar S Baslasel, Lotfi Bin Dahman, Nasher H Alyami, Abdullah Abu Bakar, Mohamed Badheeb

**Affiliations:** 1 Medicine, Hadhramout University, Mukalla, YEM; 2 Oncology, King Khalid Hospital - Oncology Center, Najran, SAU; 3 Urology, King Khalid Hospital, Najran, SAU; 4 Pathology and Laboratory Medicine, King Khalid Hospital, Najran, SAU; 5 Urology, Ibb University, Ibb, YEM; 6 Radiology, King Khalid Hospital, Najran, SAU; 7 Urology, National Institute of Urology and Nephrology Egypt, Cairo, EGY; 8 Clinical Biochemistry, College of Medicine, Hadhramout University, Mukalla, YEM; 9 Internal Medicine, King Khalid Hospital, Najran, SAU; 10 Ophthalmology, King Khalid Hospital, Najran, SAU; 11 Internal Medicine, Bridgeport Hospital, Yale New Haven Health, Bridgeport, USA

**Keywords:** transurethral resection of the bladder tumor, survival, intravesical gemcitabine, intravesical chemotherapy, sarcomatoid carcinoma, carcinosarcoma, case report, bladder cancer

## Abstract

Carcinosarcoma or sarcomatoid carcinoma of the urinary bladder is a rare but aggressive bladder cancer characterized by malignant epithelial and mesenchymal components, with only a few cases reported in the literature so far. In this report, we discuss a case of a 74-year-old female nonsmoker who presented with intermittent hematuria and passage of clots in the last four months. Radiographic images showed an irregular mass lesion (6.2 x 6 cm) in the left lateral wall of the urinary bladder near to left vesicoureteral junction. The mass was completely removed with transurethral resection of the bladder tumor (TUR-BT). Histopathological study revealed high-grade carcinosarcoma, and immunohistochemistry showed diffuse positivity for vimentin, pan-cytokeratin (CK) and CK7, epithelial membrane antigen (EMA), and CK5/6. The patient declined radical cystectomy and only agreed to receive intravesical chemotherapy (gemcitabine), and she remains alive after more than four years of follow-up.

Carcinosarcoma of the urinary bladder is a rare tumor primarily affecting older people, and it is most commonly treated with radical cystectomy and different combination treatments such as chemotherapy and radiation. However, tumor resection followed by intravesical chemotherapy may be an alternative option in the early stages of bladder carcinosarcoma for some patients, thereby avoiding the need for aggressive treatments, especially for elderly patients who decline to undergo radical surgery.

## Introduction

Urinary bladder carcinosarcoma, also known as sarcomatoid carcinoma, is a rare disease and accounts for less than 0.5% of all bladder cancers. It is distinguished by the presence of both an epithelial urothelial component and spindle cells with a sarcomatous connective appearance [1]. Carcinosarcoma's mesenchymal element lacks epithelial markers, and patients with carcinosarcoma usually present at a later stage and have a higher risk of mortality than patients with high-grade urothelial carcinoma [2,3]. Additionally, bladder carcinosarcoma cases show monoclonal origin for epithelial and mesenchymal components, with multiclonal stem cells potentially playing a causative role [2,4]. It is an uncommon bladder tumor with a male-to-female ratio of 2:1 and often appears in the seventh decade of life [1,3,5]. Due to its rarity, its behavior and treatment choices are contested. It usually appears at an advanced stage (70%), with macroscopic hematuria and dysuria being the most prevalent symptoms [1]. Urinary bladder carcinosarcomas have a terrible prognosis, with the majority of patients dying within one year of diagnosis [5]. The pathological stage is the primary predictor of survival, and regional and distant spread raises death rates [5,6]. We report the case of a 74-year-old female who presented with intermittent hematuria and passage of clots and was subsequently diagnosed with urinary bladder carcinosarcoma.

## Case presentation

A 74-year-old female nonsmoker presented with intermittent hematuria and passage of clots for the last four months that had been aggravated for 20 days accompanied by severe lower urinary tract symptoms (LUTS). The patient had no comorbidities or family history of cancer. No mass or other abnormality was detected on abdominal and digital rectal examinations. The laboratory data including complete blood cell counts and renal and liver function tests on admission were within normal limits. The urine analysis showed many red blood cells (RBCs) and urine cytology was highly positive for malignancy.

**Figure 1:**
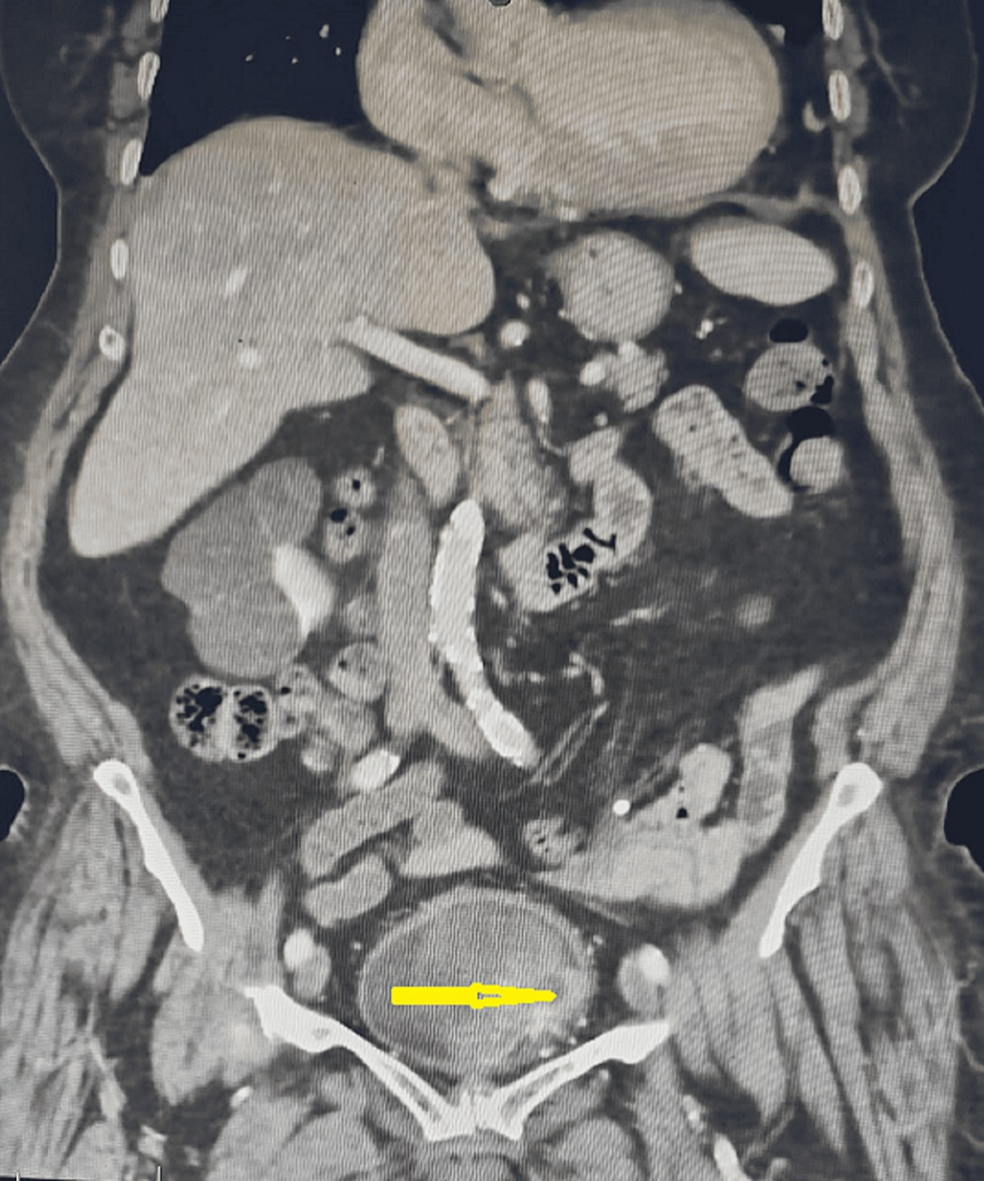
An abdominal CT scan with contrast revealed a 6.2 × 6 cm heterogeneous mass in the left wall and base of the bladder (arrow) CT: computed tomography

Abdominal ultrasonography (US) showed an irregular flat mass lesion of 5 x 3.5 cm in the left lateral wall of the urinary bladder. An abdominal CT scan showed mild wall thickening in the urinary bladder, with a large, faintly hyperdense soft tissue mass lesion, occupying the larger part of the urinary bladder in its left aspect, measuring about 6.2 x 6 cm in dimensions; it appeared heterogeneously enhancing post intravenous contrast injection with no renal hydroureteronephrosis (Figure 1).

Additionally, a metastatic workup including a CT scan of the chest and abdomen showed no metastatic lesions in the lungs and liver. Abdominal MRI of the pelvis revealed an ill-defined irregular T1 iso/T2 hypo to intermediate signal intensity mass lesion in the left lateral and posterior wall of the urinary bladder. Transurethral resection of bladder tumor (TUR-BT) was performed, which revealed a large irregular flat lesion near the left ureterovesical junction that extended from the bladder base to the dome. Complete resection was performed, and a deep muscle biopsy was taken. The patient was discharged a day after surgery without any complications.

**Figure 2:**
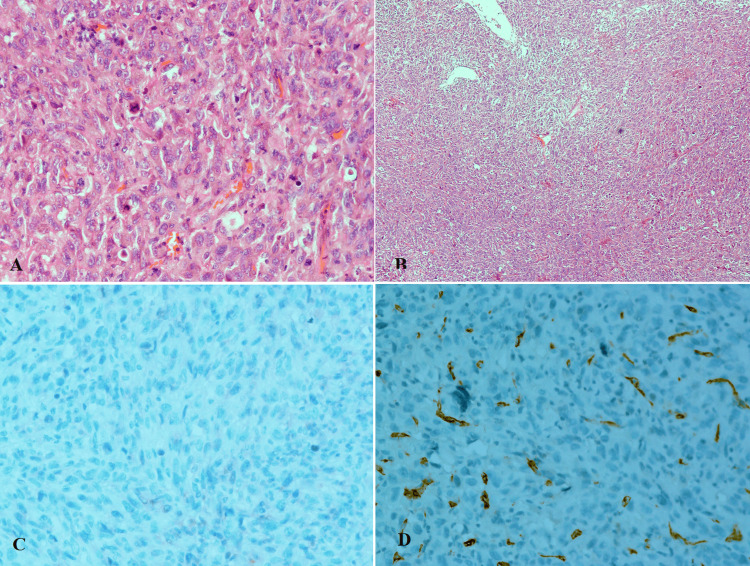
Histopathology of the resected tumor in microscopic view with epithelial and sarcomatoid components A: H & E ×20; B: H & E ×40. Immunohistochemical staining showed negativity for CK20 (C) and CD34 (D)

The histopathology report of the removed specimen showed malignant tumors composed of solid sheets of spindle and epithelioid malignant cells and rare residual glandular patterns. These cells revealed highly pleomorphic, hyperchromatic nuclei, prominent nucleoli, and plenty of granular eosinophilic cytoplasm. The tumor showed high mitotic activity and prominent necrosis (Figure 2).

**Figure 3:**
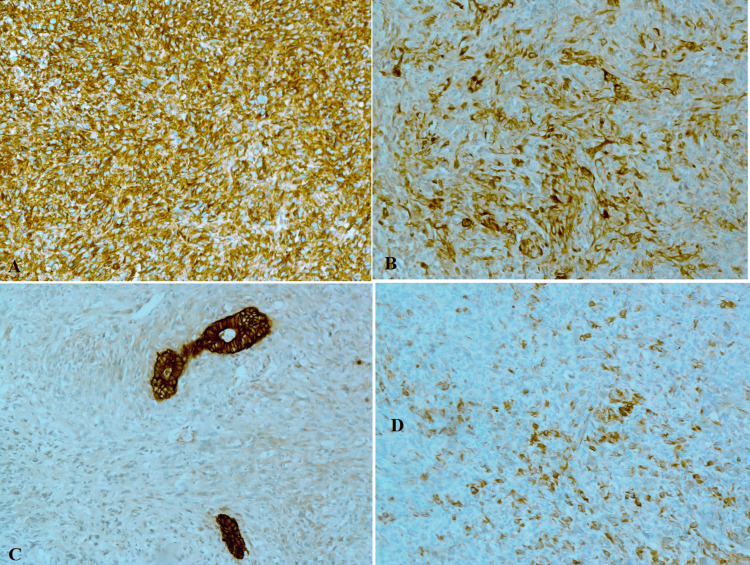
Immunohistochemical staining showing diffuse positivity for vimentin (A), pan-cytokeratin (B), CK5/6 (in the glandular foci) (C), and CK7 (D)

There was no muscle invasion, and muscularis propria was free of tumor, and the separately sent deep muscle was also free of tumor. Immunohistochemistry showed diffuse positivity for vimentin, pan-cytokeratin (CK), CK7, epithelial membrane antigen (EMA) (focally), and CK5/6 (in the glandular foci). The remaining immunostains for desmin, h-Caldesmon, HMB-45, P63, CD68, CK20, Uroplakin, CD34, LCA, Neurofilament, BCL2, and CD99 were negative (Figures 2-3). Based on pathological examination, the final diagnosis was a high-grade carcinosarcoma of the urinary bladder and the pathological stage was pT1.

**Figure 4:**
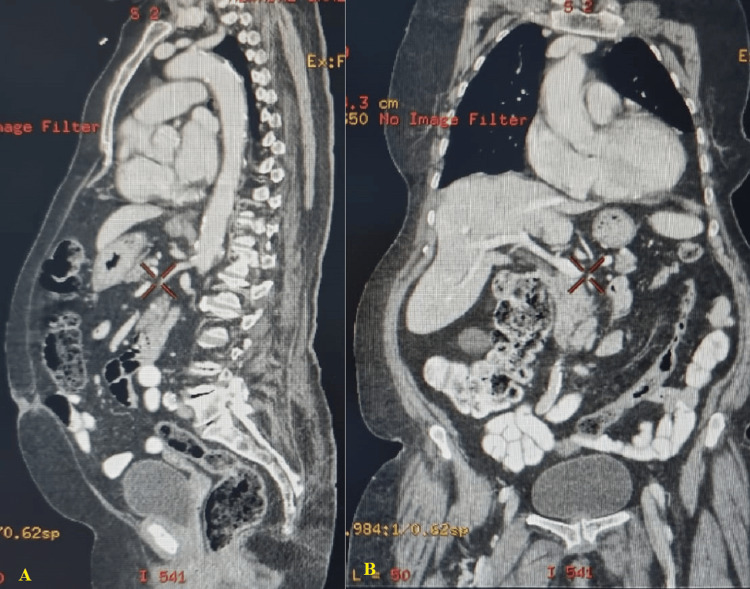
Follow-up abdominal CT scan with contrast three months after treatment revealed a normal urinary bladder without any abnormality or intraluminal lesion A: lateral view; B: anterior view CT: computed tomography

The case was discussed in the institutional tumor board meeting; the patient was given the surgical option of radical cystectomy with pelvic lymph node dissection with ileal conduit diversion. She was counseled about the advantages and disadvantages of all available modalities. Her family members were also involved in the discussion. She refused any form of surgical management because of the procedure's invasiveness. Additionally, she refused any radiotherapy or systemic chemotherapy. She was treated with intravesical gemcitabine chemotherapy [Intravesical installation once a week for six weeks at a dosage of 2,000 mg/50 mL (induction treatment), then once a month for two years (maintenance therapy)]. The patient underwent regular follow-ups with urine cytology and periodic cystoscopy every three months and radiologic imaging diagnostics such as a chest-abdominal-pelvic CT scan every three to six months. A cystoscopy three months later showed no malignancy in the urinary bladder. The patient remains alive after four years of follow-up, with no metastasis or relapse reported in cystoscopic and radiologic follow-ups (Figure 4).

## Discussion

Carcinosarcomas of the urinary bladder have an aggressive nature and are uncommon, accounting for 0.1-0.3% of all bladder malignancies [7]. Urinary bladder carcinosarcomas primarily affect older individuals, typically appearing in their seventh decade of life, with a higher prevalence in men (male-to-female ratio: 2:1) [1,3,5]. Our patient was in the seventh decade of life but was female, which contrasts with previous reports of carcinosarcomas. The etiology of carcinosarcomas is unknown; however, previous pelvic radiation therapies or systemic chemotherapy may cause bladder problems and subsequently lead to the growth of sarcomatoid carcinoma [8]. Additionally, evidence suggests a monoclonal origin for epithelial and mesenchymal components of carcinosarcomas, but genetic diversity may occur in subsequent clonal evolution [9]. Our patient was not a smoker, did not suffer from other cancers, and had not received chemotherapy or radiotherapy.

Carcinosarcomas are commonly detected around the bladder's trigone as it is hypothesized to originate from the Wolffian body [10]. According to current research, the tumor is often found on the bladder's lateral wall, and the common symptoms include hematuria, dysuria, frequent urination, and obstructive symptoms, as seen in our patients [5,7]. Due to the tumor's rarity, data in the literature are limited to case reports and limited series [8,11]. While several therapeutic techniques have been documented, the most effective treatment involves a multimodality approach [6]. The recommended treatments include TUR-BT, radical cystectomy, partial cystectomy with chemotherapy or radiotherapy, and neoadjuvant radiotherapy followed by radical cystectomy and adjuvant radiotherapy [5]. Radical cystectomy is the most effective modality, with aggressive surgery being the only curative option; however, it does not prevent local recurrence or metastasis. TUR-BT and partial cystectomy raise the possibility of inadequate tumor resection [12].

In the present case, the patient refused radical cystectomy, systemic chemotherapy, or radiotherapy. Hence, a decision was made to treat her with intravesical chemotherapy (gemcitabine) with regular follow-ups. Varshney et al.'s study has described a similar treatment modality [11]. A summary of recently reported bladder carcinosarcoma cases treated with transurethral resection is provided in Table 1 [4,13-16].

**Table 1:** A summary of recently reported bladder carcinosarcoma cases treated with transurethral resection BCG: Bacillus Calmette-Guérin; TUR-BT: transurethral resection of bladder tumor

Authors	Year	Symptoms	Age in years/sex	Treatment	Tumor size, cm	Tumor location	Outcome
Akoluk et al. [4]	2011	Gross, painless hematuria	80/M	TUR-BT × 2, radical cystectomy	2–3	Left lateral wall, in trigone	No chemotherapy or radiotherapy, alive 27 months postop
Hirano et al. [13]	2018	Gross hematuria	77/M	TUR-BT	2.5	Right bladder wall	No chemotherapy or radiotherapy, alive 27 months
Zaitsu et al. [14]	2011	Painless gross hematuria	83/M	TUR-BT × 2	10	Right wall	BCG instillation intravesically, alive 6 months postop
Hoshi et al. [15]	2007	Painless gross hematuria	58/F	TUR-BT and partial cystectomy	4.1	Right wall	Neoadjuvant chemoradiotherapy, alive 30 months postop
Kumar et al. [16]	2022	Intermittent gross hematuria and clot passing	74/M	TUR-BT	5 x 3.5	Right bladder wall, near the vesicoureteric junction	Neoadjuvant chemotherapy, died after 9 months
Our case	2020	Intermittent hematuria and clot passing	74/F	TUR-BT	6.2 x 6	Right bladder wall	Gemcitabine instillation intravesically, alive 4 years postop

Zachariadis et al. have described an intriguing case of a 76-year-old female with a history of heart failure and diabetes treated with TUR-BT followed by radiation. However, the patient did not complete the protocol due to the progression of the disease and died five months later [17]. Hirano et al. reported a 77-year-old patient diagnosed with urinary bladder carcinosarcomas and treated with TUR-BT alone and remained alive after 27 months of therapy without recurrence or metastasis [13]. Zaitsu et al. reported an 83-year-old patient diagnosed with urinary bladder carcinosarcomas and treated with TUR-BT followed by intravesical BCG instillation and was alive after six months of therapy without recurrence or metastasis [14]. However, more aggressive therapies than TUR-BT alone are usually adopted as treatments for bladder carcinosarcoma due to the invasiveness of the tumor. In general, given its aggressive nature, increased likelihood of metastasis, and poor survival, utmost care must be taken and close monitoring of radiologic and other clinical manifestations should be performed. More research and multicenter prospective studies are needed to gain deeper insights into the condition, its underlying pathophysiology, and innovative targeted therapeutics.

Urinary bladder carcinosarcomas have a terrible prognosis, with the majority of patients dying within one year of diagnosis [5]. The pathological stage is the primary predictor of survival, and regional and distant spread raises death rates [5,6]. Wang et al.'s study, which included 221 patients, found that the disease prognosis was mainly determined by the tumor stage at presentation. Individuals with regional or distant metastases had a higher risk of cancer-related death than those with localized disease. The one-, five-, and 10-year survival rates were 53.9%, 28.4%, and 25.8%, respectively, demonstrating the aggressive biological activity of sarcomatoid cancer. However, there was no significant difference in overall survival between those treated with TUR-BT and radical radiotherapy vs. radical cystectomy [8]. Adjuvant radiation and chemotherapy combinations provide varying results. However, poor treatment results in sarcomas are frequently attributed to advanced disease stages or poor patient health. Combining carboplatin and gemcitabine with irradiation has shown promising outcomes, while standard treatment frequently fails to react well to simple radiation therapy [7,17].

Our patient is still alive without any recurrence or metastasis after more than four years of follow-up. The early stage and superficial tumor involvement without submucosal infiltration in the reported cases including our case may contribute to longer survival and absence of recurrences [11,13,14]. In patients with early-stage sarcomatoid urothelial tumors, TUR-BT with adjuvant intravesical chemotherapy, such as pirarubicin, has limited effectiveness [13]. The intravesical chemotherapy with gemcitabine, as in our case, may reduce the chance of recurrence as the sarcoma is sensitive to this chemotherapy. However, given the tumor's aggressive nature, radical treatment should be pursued whenever possible, and radical cystectomy should be pursued in fit persons with surgically resectable tumors [9].

## Conclusions

Carcinosarcoma of the urinary bladder is a rare tumor primarily affecting older people. This condition is commonly treated with radical cystectomy and different combination treatments such as chemotherapy and radiation. However, tumor resection followed by intravesical chemotherapy may be an alternative option for some patients in the early stages of bladder carcinosarcoma, obviating the need for aggressive treatments, specifically for elderly patients who decline radical surgery. Further studies need to be conducted to identify effective treatment protocols for this patient population.

## References

[1] Althubiany HH, Hasan RM, Alzahrani SA, Bahdilh S (2020). Case report of a rare urinary bladder tumor variant (carcinosarcoma). Urol Ann.

[2] Halachmi S, DeMarzo AM, Chow NH (2000). Genetic alterations in urinary bladder carcinosarcoma: evidence of a common clonal origin. Eur Urol.

[3] Wright JL, Black PC, Brown GA, Porter MP, Kamat AM, Dinney CP, Lin DW (2007). Differences in survival among patients with sarcomatoid carcinoma, carcinosarcoma and urothelial carcinoma of the bladder. J Urol.

[4] Akoluk A, Barazani Y, Slova D, Shah S, Tareen B (2011). Carcinosarcoma of the bladder: case report and review of the literature. Can Urol Assoc J.

[5] Basibuyuk I, Topaktaş R, Elbir F (2017). Bladder carcinosarcoma: a case report with review of the literature. Arch Ital Urol Androl.

[6] Kouhen F, Dahbi Z, Afif M (2023). Bladder carcinosarcoma treated by cystectomy and adjuvant chemotherapy with good outcomes: a case report. J Med Case Rep.

[7] Wallach JB, Wang B, Sanfilippo N (2009). High-grade hyperinvasive sarcomatoid urothelial bladder carcinoma demonstrating complete response to bladder-preserving chemoradiation. Curr Oncol.

[8] Wang J, Wang FW, Lagrange CA, Hemstreet Iii GP, Kessinger A (2010). Clinical features of sarcomatoid carcinoma (carcinosarcoma) of the urinary bladder: analysis of 221 cases. Sarcoma.

[9] Tiwari RV, Ngo NT, Lee LS (2020). The optimal management of variant histology in muscle invasive bladder cancer. Transl Androl Urol.

[10] Atılgan D, Gençten Y (2013). Carcinosarcoma of the bladder: a case report and review of the literature. Case Rep Urol.

[11] Varshney B, Choudhary GR, Yadav T, Nalwa A (2021). Sarcomatoid urothelial carcinoma of the bladder with osseous metaplasia: bone in urinary bladder. BMJ Case Rep.

[12] Xiao J, Chen H, Ge J, Liu T (2024). Clinical efficacy analysis of partial cystectomy and radical cystectomy in the treatment of muscle-invasive sarcomatoid carcinoma of the urinary bladder. Front Oncol.

[13] Hirano D, Yoshida T, Funakoshi D, Sakurai F, Ohno S, Kusumi Y (2018). A case of early stage bladder carcinosarcoma in late recurrence of urothelial carcinoma after transurethral resection. Case Rep Urol.

[14] Zaitsu M, Yamanoi M, Mikami K, Tonooka A, Uekusa T, Takeuchi T (2011). A case of giant bladder carcinosarcoma without submucosal invasion. Case Rep Med.

[15] Hoshi S, Sasaki M, Muto A (2007). Case of carcinosarcoma of urinary bladder obtained a pathologically complete response by neoadjuvant chemoradiotherapy. Int J Urol.

[16] Kumar Pal A, Srinivas BH, Kalra S, Dorairajan LN, Sreenivasan Kodakkattil S (2022). Sarcomatoid urothelial carcinoma of the urinary bladder with chondrosarcomatous and concurrent divergent squamous cell carcinoma differentiation - a rare entity. Cureus.

[17] Zachariadis C, Efthimiou I, Giannakopoulos S, Bantis A, Giatromanolaki A, Sivridis E, Touloupidis S (2011). A case report of urinary bladder carcinosarcoma and review of the literature. Case Rep Urol.

